# In Vivo and In Vitro Studies of Cigarette Smoke Effects on Innate Responses to Influenza Virus: A Matter of Models?

**DOI:** 10.3390/v14081824

**Published:** 2022-08-20

**Authors:** Wenxin Wu, Jeremy S. Alexander, Jordan P. Metcalf

**Affiliations:** 1 Pulmonary, Critical Care & Sleep Medicine, Department of Medicine, the University of Oklahoma Health Sciences Center, Oklahoma City, OK 73104, USA; 2 Department of Microbiology and Immunology, the University of Oklahoma Health Sciences Center, Oklahoma City, OK 73104, USA; 3 Veterans Affairs Medical Center, Oklahoma City, OK 73104, USA

**Keywords:** cigarette smoke, influenza, viral infection, antiviral immunity, innate immune evasion

## Abstract

Cigarette smoke (CS) is a significant public health problem and a leading risk factor for the development of chronic obstructive pulmonary disease (COPD) in the developed world. Respiratory viral infections, such as the influenza A virus (IAV), are associated with acute exacerbations of COPD and are more severe in cigarette smokers. To fight against viral infection, the host has developed an innate immune system, which has complicated mechanisms regulating the expression and activation of cytokines and chemokines to maximize the innate and adaptive antiviral response, as well as limiting the immunopathology that leads to exaggerated lung damage. In the case of IAV, responders include airway and alveolar epithelia, lung macrophages and dendritic cells. To achieve a successful infection, IAV must overcome these defenses. In this review, we summarize the detrimental role of CS in influenza infections. This includes both immunosuppressive and proinflammatory effects on innate immune responses during IAV infection. Some of the results, with respect to CS effects in mouse models, appear to have discordant results, which could be at least partially addressed by standardization of animal viral infection models to evaluate the effect of CS exposure in this context.

## 1. Introduction

Influenza A Virus (IAV) infection is a major cause of infectious morbidity and mortality [1]. The virus has a single-stranded RNA (ssRNA) genome composed of eight linear segments and belongs to the Orthomyxoviridae family [2]. In the initial stage of virus replication, the hemagglutinin (HA) glycoprotein binds to specific sialic acid (SA) receptors on the surface of targeted cells. Then, virus particles enter the cell through receptor-mediated endocytosis [3]. Once inside the cells, the influenza virus shuts off host cell protein synthesis and replicates quickly and efficiently. Due to minor genetic variations, surface glycoprotein structures are modified in a process called antigenic drift, while an exchange of whole gene segments occurs during coinfection of a host species, which is known as antigenic shift. The viral genetic changes, including antigenic drift and antigenic shift, decrease the efficacy of vaccination, thereby causing yearly epidemics and, in the case of antigenic shift, causing large-scale pandemics with millions of deaths, such the 1918 Spanish Flu [4].

The body’s frontline defense against both viral and microbial pathogens is the innate immune system, which is composed of a number of signaling cascades that initiate responses to infections [5]. The innate immune responses to IAV are triggered by recognition of pathogen-associated molecular patterns (PAMPs) by the host’s internal or cell surface pattern-recognition receptors (PRRs). PRRs are critical for recognition of IAV and initiation of early antiviral innate immune responses, which include the detection of virus genetic material, the induction of antiviral cytokine responses, and the triggering of inflammation, in order to contain the viral infection [6]. Three classes of PRRs have been identified for IAV: Retinoic acid-inducible protein I (RIG-I), nucleotide-binding oligomerization domain 2 (NOD2), and Toll-like receptors (TLRs) [7].

RIG-I, which is expressed in most cell types, is involved in the detection of IAV infection by the recognition of viral ssRNA. The 5’-triphosphorylated end of ssRNA is bound by RIG-I [8]. After recognition of IAV, RIG-I recruits the mitochondrial antiviral signaling protein (MAVS) and initiates a signaling cascade, including transcription factors called interferon regulatory factors (IRFs) and the nuclear factor kappa-light-chain-enhancer of activated B cells (NF-κB) [9]. Endosomal TLRs are found within the endosome of cells [8]. TLR3, a double-stranded RNA sensor, is used by some epithelial cells to detect the viral replicative intermediate double-stranded RNA (dsRNA) [10,11]. TLR7 is expressed mostly in plasmacytoid dendritic cells (pDC) and recognizes AU rich sequences of IAV ssRNA [12]. Immune cells are the primary expressers of NOD2 and NLR family pyrin domain-containing 3 (NLRP3). Both are involved with IAV infection recognition [9].

Induction of interferon (IFN) is a critical component of the host innate response to influenza infection. IFNs are further divided into type I (mainly IFN-α and β), type II (IFN-γ) and type III (IFN-λ) subtypes, based in part on the differential use of unique receptors through which they mediate signal transduction to induce antiviral activity [13]. Type I and type III IFNs are bound by different receptors but have similar downstream signaling pathways. IRFs are essential for the induction of IFNα/β and IFN-λ, while NF-κB is thought to be required as a co-factor [5]. Signaling of immune responses, suppression of viral replication, and expression of additional IFN are propagated to nearby cells by the activation of IFN receptors. Inflammation is also a component of the body’s innate response to infection [14]. Proper function of inflammation is to eliminate the virus, clear out necrotic cells and tissues damaged from the original infection, and initiate tissue repair. During inflammation, leukocytes are recruited from the blood into the lung by proinflammatory cytokines and chemokines such as IL-1β, tumor necrosis factor alpha (TNF-α), interleukin 8 (IL-8), IL-12, and interleukin 6 (IL-6) [7,15]. Leucocytes are also recruited to the site of infection by TNF-α-mediated vasodilation and enhanced leucocyte adhesion [16]. Neutrophils are recruited to the area of infection by detecting chemical gradients of molecules, such as IL-8, IFN-γ, C3a, C5a, and Leukotriene B4. As part of the inflammatory response, armed effector T cells are recruited to the site of infection to initiate the adaptive immune response [17]. As is the case with other biological processes, inflammation can be harmful when it is prolonged or disproportionate. Disordered inflammation contributes to conditions including chronic obstructive pulmonary disease (COPD), autoimmune disease, and cancer [14,18,19]. Viruses are detected during half of all COPD exacerbations, and are associated with worse clinical outcomes. Human rhinovirus (HRV), respiratory syncytial virus, and IAV are the most frequently detected viruses during exacerbation [20]. Furthermore, recurrent exacerbations of COPD, mainly caused by viruses, increase disease progression and mortality, likely due to enhanced airway inflammation [21].

Cigarette smoke (CS), either as direct exposure or second-hand smoke, has been connected to many health issues. Along with its carcinogenic effect, CS has been linked with cardiovascular disease and pulmonary disease. The pulmonary risks include an increased chance of infection, increased severity, and increased chance of hospitalization with IAV [22,23,24]. CS exposure increases the frequency and severity of respiratory tract viral infections [25,26,27]. It is well known that CS is composed of many chemical components that have various effects on the human body [28,29]. This summary will provide a panoramic review on how CS affects innate immune responses and IAV infection outcomes using models including in vitro cell culture and in vivo animal smoking.

## 2. In Vitro Cell Culture Studies

### 2.1. Epithelial Cells and Immune Leukocytes in the Innate Immune Response to IAV

The most common route of transmission for respiratory pathogens is via the upper and lower airways. Epithelial cells are the primary site of viral replication for influenza, although monocytes/macrophages and other leukocytes can also be infected [30,31]. The innate immune response is triggered in epithelial cells, alveolar macrophages, and dendritic cells (DC), leading to production of antiviral IFN and proinflammatory cytokines and chemokines [31,32,33].

Airway epithelial cells orchestrate the innate and adaptive immune responses to pathogens. Epithelial cells express PRRs and release antiviral type I and III IFNs and proinflammatory cytokines like TNF-α, IL-1β, IL-6, CCL2, CCL5, IL-8, and IP-10 into the airways, which recruit subsets of leukocytes and T cells that are appropriate for inflammatory and immune responses to contain viral spread [34,35,36]. They also interact with DC to alter antigen presentation to T cells [37].

Airway immune cells are also vitally important in detection and elimination of invading microbes. The leukocytes, such as macrophages, monocytes, neutrophils, DCs, eosinophils, and natural killer cells, are activated in response to IAV to eliminate the virus, trigger wound repair, and produce more chemokines and cytokines such as MCP-1, IL-6, and IL-8 [38,39]. Plasmacytoid DCs also produce high levels of type I IFNs by TLR7 recognition of IAV and via IRF7 activation [40]. Innate immune responses to IAV infections in the respiratory tract are further reviewed in [41].

### 2.2. CS Effects on Epithelial Cells during Viral Infection

Cigarette smoke extract (CSE) has been widely used for in vitro and in vivo experiments to study smoking effects. Most cell culture studies are carried out with CSE treatment followed by viral infection. CSE is a solution composed of all the aqueous constituents of common (directly inhaled) CS. For a review on CSE preparation, see Gellner et al. [42]. Typically, one cigarette without a filter is combusted with a pump and the smoke is bubbled through a cell culture medium at a certain speed. The resulting suspension is filtered through a 0.22-µm pore filter to remove bacteria and large particles. A fresh preparation of this solution is diluted and applied to cell cultures.

In general, CSE has immunosuppressive effects on the IAV infection-induced innate immune response. Using human precision-cut lung slices (PCLS) and human primary epithelial cells, it has been shown that antiviral responses are reduced by CSE [43]. Specifically, RIG-I, TLR3, IP-10, IFN-λ, and IFN-β induction by IAV were all decreased by CSE in cultured epithelial cells [44]. In addition, smoking reduces the expression of antiviral cytokines in primary respiratory epithelial cells isolated from active smokers [45,46]. It has been demonstrated that a dose-dependent decrease in human lung epithelial cell antiviral responses against a viral double-strand RNA (dsRNA) mimic, poly I:C, in the presence of CSE. Mechanistically, CSE decreases the expression of IFN-stimulated gene 15 (ISG15) and IRF-7 transcripts, and suppresses the nuclear translocation of key transcription factors, NF-κB and IRF-3, after poly I:C stimulation [47].

Recently, using lung epithelial cells or human PCLS cultured at air–liquid interface (ALI) exposed to CS or air, two research groups showed that IAV induced activation of TLR3 and secretion of antiviral cytokines (IFN-α2a, IFN-λ, IP-10), proinflammatory cytokine IL-6 and T cell-associated cytokines were completely suppressed after smoke exposure [48,49].

Human rhinovirus (HRV), which is responsible for more than half of common cold illnesses, is the other well-researched viral pathogen for epithelial cell infection responses [50]. Epithelial cells infected with HRV produce numerous chemokines, cytokines, and host defense molecules [51]. CSE increased HRV-induced TLR3 expression and HRV-induced IL-8 secretion at lower concentrations in A549 cells. On the contrary, CSE treatment inhibited HRV-induced IL-6 responses in a dose-dependent manner [52]. Inhibition of HRV-induced IP-10 by CSE was mediated, at least in part, via transcriptional regulation. CSE induced a concentration-dependent decrease of IP-10 and IFN-β responses to HRV in airway epithelial cells [53]. Further analysis found that CSE reduces chromatin accessibility and inhibits viral signaling via NF-κB, IRF-1, STAT-1, and MDA5 [54].

Similarly, CSE significantly suppresses many HRV-induced genes, which are associated with antiviral defenses, inflammation, viral signaling, and airway remodeling [55]. The same group showed that, although CSE treatment alone induced approximately 2500 statistically significant gene expression changes, the most highly induced genes were associated with metabolism and/or redox pathways, or with iron binding [55]. In terms of innate responses, IL-8 is the only proinflammatory cytokine that is released more after CSE treatment [56]. The mechanism of this effect was shown by another group to be occurring through IL-8 mRNA stabilization [51,57]. Only one report found that the combined exposure of CSE and bacterial lipopolysaccharide (LPS) was associated with increase in the release of cytokines MCP-1 and IL-6 in addition to IL-8 [58]. CSE has also been shown to directly inhibit NF-κB expression in human airway smooth muscle cells [59].

One of the most important concepts in CS-enhanced respiratory viral infections is that CS continues to be the leading risk factor for acute exacerbations of COPD in the developed world, which are associated with respiratory viral infections [60]. Some of the most translationally relevant studies on this topic use primary cells from COPD patients or smokers. Mallia et al. used experimental HRV infection in subjects with COPD and in control subjects [61]. They found that type I and type III IFN production by bronchoalveolar lavage (BAL) cells was impaired in response to HRV in the subjects with COPD. Subjects with COPD developed neutrophilic inflammation that was greater and more prolonged than with the control group. They speculated that impaired IFN production and neutrophilic inflammation may be important mechanisms in virus-induced COPD exacerbation. Our own data also demonstrated that human primary airway epithelial cells isolated from active smokers have impaired antiviral responses, including RIG-I, TLR3, and IFNs, to IAV infection [44]. One report found that epithelial IFN response in cells from donors with COPD was delayed and that maximal IFN production did not occur until 72–96 h post-infection in COPD patients. Thus, they proposed that viral exacerbations of COPD were related to the delayed, rather than deficient, induction of antiviral genes, which leads to a delayed and exaggerated inflammatory host immune response [62].

### 2.3. CS Effects on Immune Leukocytes during Viral Infection

In pDCs, in the absence of viral infection, CSE alone has the potential to diminish antiviral immunity by downregulating the release of IFN-α, TNF-α, and IL-6, while at the same time augmenting IL-8-induced recruitment of neutrophils, which may have other effects [63]. Oxidative stress generated by CSE treatment suppresses the generation of cytokines IL-12 and IL-23 by human monocyte-derived DCs through the activation of ERK-dependent pathways [64].

CS attenuation of poly I:C-induced innate antiviral responses in human peripheral blood mononuclear cells (PBMC) is mainly due to inhibition of IFN-β production. A marked attenuation of IRF-3 and NF-kB activation occurs in poly I:C-stimulated PBMCs exposed to CSE. Similarly, PBMCs from smokers and smoke-exposed mice also displayed marked reduction of poly I:C-induced antiviral responses compared with either non-smokers or sham-exposed mice. In the same study, CS was found to block the production of type I IFNs following poly I:C treatment, and inhibited subsequent STAT1 activation [65].

In alveolar macrophages, CSE exposure suppressed TLR-induced TNF-α, IL-6, IL-10, and Regulated on Activation, Normal T cell Expressed and Secreted (RANTES) production, but had no effect on IL-8 production [66]. Active smoking also reduces both the proportion of human lung macrophages expressing TLR3, and dsRNA-induced IP-10 production [67].

Horvath and colleagues found that CS affects epithelial–DC crosstalk. Nasal epithelial cells (NECs)/mono-DC co-cultures, using NECs from smokers, exhibited suppressed induction of the T-cell/natural killer cell chemokine IP-10 after infection with IAV, indicating that NECs from smokers may skew early IAV-induced type 1 T helper (Th1) responses. NECs isolated from smokers modify the responses of DCs by developing a cytokine microenvironment that suppresses the IFN-mediated Th1 response and enhances the Th2 response [45].

Again, as in epithelial cells, there are few reports showing that CS increases innate response in immune leukocytes. One report found that TLR3-positive alveolar macrophage numbers were significantly increased in smokers compared with non-smoking control subjects. Furthermore, CSE potentiated the expression of TLR3 in monocyte-derived macrophages and significantly augmented the release of IL-8, as well as total matrix metalloproteinase (MMP)-9 activity, in cells treated with a TLR3 ligand [68]. CS decreases production of many cytokines from human pDCs while promoting the release of IL-8 in response to TLR-9 stimulation [63].

Altogether, using PAMP or live viruses as agonists, in vitro studies demonstrate that CSE suppresses the IAV-induced immediate-early and amplification phases of the type I IFN, as well as proinflammatory cytokine production, except for the release of IL-8. Suppression of IFN may lead to impaired antiviral responses against IAV, thereby increasing the risk and consequences of airway infections. Contrastingly, increased release of IL-8 may lead to a magnified recruitment of neutrophils into the lungs. Aside from these antiviral changes during viral infection, CS alone induces injury to the airway epithelium and promotes epithelial remodeling, resulting in impaired mechanical defenses against infection by decreasing mucociliary clearance and disrupting barrier function [60].

## 3. In Vivo Animal Smoking Studies

Experimental animal models may help us understand how CS affects the exacerbation of viral infections and contributes to increased infection and severity in vivo [23]. In a typical whole-body smoking model, mice are placed in an exposure chamber. The chamber is attached to a cigarette-smoking machine and the mice’s whole bodies are exposed to mainstream CS generated from cigarettes once or twice per day. Mice are exposed five days per week for a varying number of weeks. Control mice are placed inside an identical exposure chamber but receive only free air.

Generally, CS exposure increases the weight loss and mortality caused by IAV infection [69,70,71]. However, animal experiments show that CS has both proinflammatory and immunosuppressive effects, depending on smoking length, smoking dose, virus dose, IAV strain, and sample collection time (Table 1). As an example, the length of CS exposure in mouse models may be as short as 3 days or as long as 6 months prior to IAV infection [72,73].

After smoking without IAV infection, CS alone tends to induce inflammation characterized with neutrophil infiltration into the mouse lung [72,75,80,84].

For short-term smoking (<3 weeks) followed by IAV infection, CS amplifies airway inflammation, damage to lung permeability, and mucus hypersecretion [82,85,91]. Mice exposed to smoke and then to influenza had more macrophages, neutrophils, and total lymphocytes in BALF at day 3 [77]. However, one group found that combined CS exposure and IAV infection resulted in lower total cell numbers, lower macrophage numbers, and lower neutrophil numbers in BALF than in the non-CS IAV infection group, although KC, IL-6, TNF-α, IL-1β, and IFN-γ were higher in the BALF in the CS IAV group after 10 days of smoking [87].

For long-term smoking (>3 weeks) followed by seasonal IAV infection, CS exposure suppresses RIG-I induction and antiviral IFN and IP-10 cytokine responses in the lung [70,81]. The proinflammatory IL-6 and TNF-α induction by IAV were first suppressed until day 4, but were then elevated by day 7. For more pathogenic 2009 pandemic H1N1 (pdmH1N1) or avian H9N2 virus infection, CS-exposed mice had significantly less weight loss and lower mortality than the control mice, which were associated with decreased production of inflammatory cytokines and chemokines, less macrophage, neutrophil, CD4+, and CD8+ T cell infiltration, and reduced lung damage [84]. Nevertheless, both short-term and long-term smoking followed by IAV infection altered the innate response, and altered pulmonary cellular recruitment and proinflammatory cytokine profiles particularly. CS may have differential effects on these responses during infection with seasonal versus pandemic viral strains.

Using the same smoking-exposure duration of three months, Robbins et al. demonstrated that CS differentially affects airway inflammatory responses depending on the initial infectious dose. Specifically, CS exposure attenuated the airway’s inflammatory response to low-dose infection, but increased inflammation with high-dose influenza [74]. To some extent, the results may explain why we have seen both immunosuppressive and proinflammatory effects by CS in animal experiments. When the mice are first infected with IAV, the amount of virus present is relatively low and CS exposure attenuates the inflammatory response. With further viral replication after days of infection, the dose becomes relatively higher in the host and CS exposure increases lung injury and inflammation at this stage.

Therefore, the sample collection time may contribute to discordant results in in vivo studies. In a multiple-sample collection model, mice pre-exposed to smoke were infected with IAV and continued to receive smoke exposure until they were killed at different time points up to day 30. There is a large difference in terms of immune cells in the BALF before and after day 9 post-infection [89]. In CS-exposed, IAV-infected mice, white blood cell and lymphocyte numbers were similar before day 9 but much higher after day 9. Macrophage numbers in these mice were lower before day 9 but higher after day 9 post-infection. Our group also reported that prior CS exposure caused a biphasic T cell and IFN-γ response to subsequent infection with influenza in the lung. Although long-term CS exposure suppressed early pulmonary IAV-antigen specific CD8+ and CD4+ T cell numbers and IFN-γ production in response to IAV infection on day 7 post-infection, CS enhanced the numbers of these cells and IFN-γ production on day 10 [90]. This is interesting, as it accords with the studies in humans that the percentage of CD8+ T lymphocytes increased in the lungs of patients at the onset of acute exacerbations of COPD, and that CD8+ T cells in COPD exhibited greater expression of cytotoxic proteins [92].

Our recent publication showed that in CS-exposed mice, early IFN-β administration significantly increased survival during IAV infection, while late IFN-β administration did not affect mortality [93]. In contrast, in non-smoking mice, both early and late IFN-β administration decreased the survival rate of mice infected with IAV. Moreover, type I IFN treatment, especially late IFN, promoted supernormal proinflammatory responses in IAV-infected non-smoking mice and recruited additional granulocytes and monocytes to the lung, which likely contributed to worsened disease outcomes. On the contrary, early IFN administration to CS-exposed mice enhanced the CS-impaired host innate antiviral response to IAV infection and decreased mortality. This study also indicated that IFNs are induced less in smokers in the early stages of IAV infection.

Taken together, studies of animal models have shown that worsened infection outcomes may be due to CS suppression of the antiviral response at an early stage of infection, but that CS may also cause an exaggerated inflammatory response, resulting in more detriment and an elongated recovery later on in IAV infection. Ideally, to put these results in context, a standard smoking model should be developed to compare all of the in vivo results. This would be aided by titrating the dose of CS exposure in mice to be equivalent to that of typical human smokers using blood cotinine levels.

## 4. Summary

**Figure 1:**
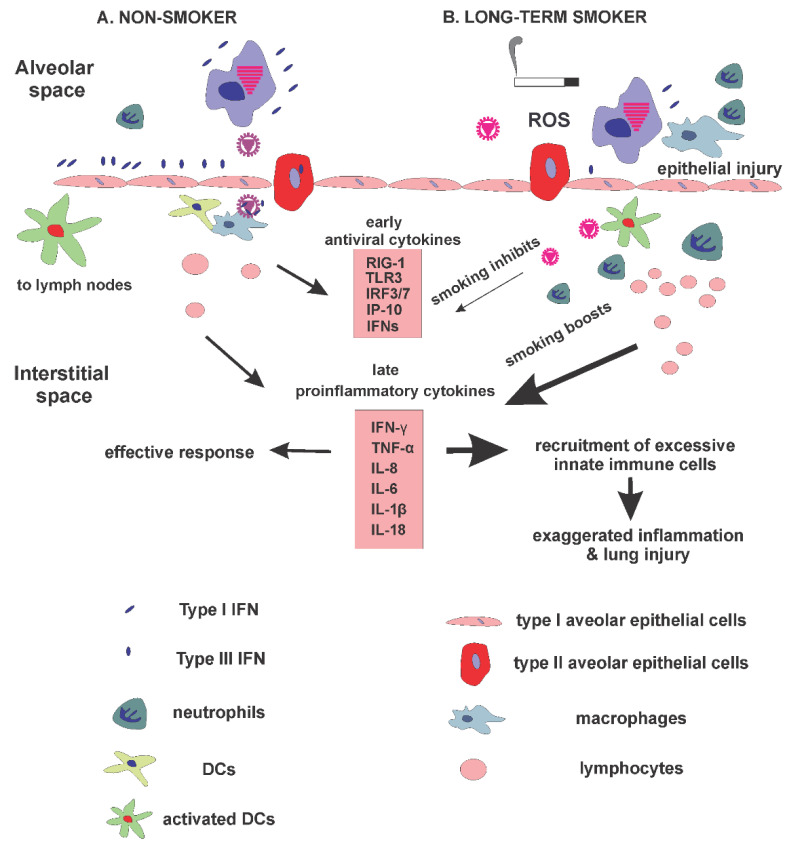
Cigarette smoking changes host innate responses during influenza virus infection. (**A**) Influenza virus-induced innate response in non-smokers. Normal responses are an exquisitely balanced biological control mechanism. (**B**) Influenza virus-induced innate response in long-term smokers. CS potentially inhibits antiviral PRR responses and cytokine induction while it boosts inflammatory cytokine induction and enhances lung injury.

Innate immune responses must be tightly regulated to maximize viral clearance while inflicting minimal damage to host cells [13]. Studies in cell cultures and animal models have revealed that CS plays a harmful role in host defenses against IAV. In many cases, CS simply disrupts the normal immune balance that usually controls the infection while limiting inflammation, by suppressing PRRs and IFN responses at an early stage, by impairing control of the infection and by promoting inflammatory immune responses enhancing injury (Figure 1). Many of the results regarding CS effects in mouse models present discordant findings, some of which could be resolved by standardization of the animal smoking models, as these discrepancies could be due to differences in viral doses and sampling times (as discussed above). The human immune response has a long history of responding to emerging respiratory viral infections, sometimes appropriately and, in the case of COVID-19, sometimes inappropriately. As long as people continue to use cigarettes, it will be important to elucidate the mechanisms underpinning the synergetic effects between viral infection and CS exposure on the activation of different signaling pathways to trigger exacerbations in COPD patients. This is not to discount the role of viral mutations in the enhancement of lung infections, as observed in SARS-CoV-2 and its variants. Thus, vaccine prevention alone is inadequate. Expansion of our knowledge with airway and lung innate immunity is necessary to improve prevention and treatment strategies for IAV infection, particularly in high-risk groups such as cigarette smokers. The studies required to achieve this goal will not only advance our understanding of the pulmonary host response against viral pathogens, but may also be directly applicable to other commonly encountered inflammatory conditions within the lung, including tuberculosis, asthma, and COPD.

## Figures and Tables

**Table 1:** Summary of in vivo animal smoking and viral infection studies.

Lead Author/Year	Smoking Length	Stimulation	Collection Time	CS Effects	Note
Thatcher 2005 [72]	1 h, twice per day for 3 days	none	N/A	inflammation↑, neutrophils ↑, neutrophil chemotactic chemokines MIP-2 and KC ↑	
Robbins 2006 [74]	2 cigarettes/d, 5 d/wk, for 3–5 months	low dose or high dose IAV H1N1 (A/FM/1/47)	Day 3, 5, and 7 after infection	inflammation with low-dose infection ↓, inflammation with high-dose influenza ↑	
Kang 2007 [75]	twice per day, 5 d/wk. for 2 wks, 1 month, or 2 months	none	N/A	IL-18, caspases 1 and 11 ↑, inflammation and emphysema ↑	
Kang, 2008 [76]	3 cigarettes/d for 2 weeks	poly (I:C), IAV H1N1 (A/PR8/34)	Day 3, 9, and 15 after infection	pulmonary inflammation and injury ↑ production of IL-18, IL-12/ IL-23p40, IFN-γ, and type I IFNs ↑	
Gualano 2008 [77]	9 cigarettes/d for 4 days	IAV H3N1 (Mem71)	Day 3 and 10 after infection	virus titers ↑ macrophages, neutrophils and total lymphocytes ↑	
Gaschler 2008 [78]	twice daily, 5 d/wk for 8 weeks	ex vivo stimulation poly (I:C) and LPS CpG	2, 6, 24 h after stimulation	TNF-α, IL-6 and RANTES ↓, nuclear translocation NF-κB ↓, AP-1 ↑	
Motz 2010 [79]	4 h/d, 5 d/wk, for 2, 8 24 weeks	ex vivo stimulation poly (I:C), ssRNA40, or ODN1826 (TLR9 agonist)	20 h after stimulation	NK cell-derived IFN-γ ↑	No difference at 2 wks; the difference emerged after 8 wks of CS exposure
Feng 2011 [69]	2h, twice daily, 5 d/wk for 6 weeks	IAV A/PR8/34	Day 7 after infection	weight loss ↑, pulmonary T-cell response ↓	
Botelho 2011 [80]	50 min, twice daily, either 4 days or 8 weeks	none	N/A	IL-1R1-dependent neutrophilia↑	
Wu 2014 [81]	2h, twice daily, 5 d/wk for 6 weeks	IAV A/PR8/34	Day 7 after infection	lung inflammation by CS alone↑ RIG-I, IFNs and IP-10 ↓ IL-6 and TNF-α ↑	
Yageta 2014 [82]	10 cigarettes daily for 4 days	IAV A/PR8/34	Day 7 after infection	pulmonary inflammation and injury ↑	
Wortham 2012 [83]	4 h/d, 5 d/wk for 6 months	IAV H3N2 (HKx31)	Day 4 after infection	NK cell hyperresponsiveness ↑ pulmonary inflammation ↑ viral clearance =	
Han 2014 [84]	2 h per episode, 2 episodes/d for 21 days	IAV H1N1 (pdmH1N1) and H9N2 (H9N2/G1)	Day 1, 3, and 5 after infection	lung inflammation by CS alone ↑. With IAV, inflammatory cytokines and chemokines ↓, macrophages ↓, neutrophils ↓, T cell infiltration ↓and lung damage ↓	
Kearley 2015 [85]	twice daily for 4 days (most data shown) or 5 d/wk for 8 and 16 weeks	IAV H1N1 (A/FM/1/47-MA)	Day 7 and 11 after infection	IFN-α, IL-6, TNF-α, IFN-γ↑ via IL-33 ↑	
Wang 2015 [86]	1 cigarette twice/d smoked for 1, 3 and 5 months.	IAV A/PR8/34	Day 1, 7, and 14 after infection	KC mRNA highest at 1 month after CS alone. KC protein highest at Day 7 after infection. IgA responses ↓	
Wang 2015 [71]	1 cigarette twice/d smoked for 1 (most data shown), 3 and 6 months	IAV A/PR8/34	Day 7 after infection	retinoic acid (RA) signaling ↓	
Bucher 2016 [87]	4 cigarettes/d for 10 days	IAV A/PR8/34	Day 5 after infection	total cells in BALF Macrophage, neutrophil ↓ KC, IL-6, TNF-α, IL-1β, IFN-γ ↑	
Mebratu 2016 [88]	6 h/d,5 days/wk, for 4 weeks	IAV HKx31 RSV, poly(I:C)	Day 14 after infection	inflammation was characterized by macrophages, lymphocytes, and neutrophils ↑	
Wang 2017 [70]	2 h, twice daily, 5 d/wk for 6 weeks	IAV A/PR8/34	Day 2, 4, and 6 after infection	weight loss ↑ lung inflammation by IAV =, RIG-I, IFN-β and IP-10 ↓ IL-6 and TNF-α before day 4 ↓, Day 6 =	
Hong, 2017 [71]	4 cigarettes/d, 5 d/wk, for 3 months	IAV H3N2 (A/Hong Kong/8/68)	Day 15 after infection	IL-17A, TNF-α, IL-6, and KC ↑, type I/II IFNs, Granzyme b, Ccl3, MIP-1α MIP-1β, and RANTES ↓	
Lee 2018 [89]	3 cigarettes/d, for 2 wks + 30 days	IAV A/PR8/34	Until Day 30 after infection	weight loss↑ neutrophils ↑ lung fibrosis↑ WBC and lymphocytes before Day 9 =, after Day 9 ↑ macrophages before Day 9 ↓, after Day 9 ↑	There is a difference before and after Day 9
Danov 2020 [48]	6 cigarettes/d for 3 days, followed by 24 cigarettes/d for the remaining 21 days	poly(I:C) and Ex vivo stimulation with IAV H1N1 (pdmH1N1)	Unknown	inflammatory DCs ↑ disrupted epithelial barrier functions↑, antiviral immune response↓	
Wu 2021 [90]	2 h, twice daily, 5 d/wk for 6 weeks	IAV A/PR8/34	Day 7 and 10 after infection	IAV-specific T cell, IFN-γ and total protein in BALF at Day 7↓, at Day 10 ↑	There is an opposite effect of CS on T cell responses to IAV at Day 7 and 10 after infection
Ferrero 2021 [91]	12 cigarettes daily for 12 days	IAV A/PR8/34	Day 5 after infection	airway obstruction, neutrophil infiltration ↑	

Notes: h = hour(s); d = day(s); wk = week. “↑” means increased; “↓” means decreased; “=” means no change.
